# Dietary Proteins and Angiogenesis

**DOI:** 10.3390/nu6010371

**Published:** 2014-01-17

**Authors:** Miguel Ángel Medina, Ana R. Quesada

**Affiliations:** 1 Department of Molecular and Biochemistry, University of Málaga, Málaga E-29071, Spain; E-Mail: quesada@uma.es; 2 CIBER de Enfermedades Raras (CIBERER), Málaga E-29071, Spain

**Keywords:** dietary proteins, angiogenesis, lactoferrin, lactadherin, angiogenin-2, lactoferricin, food lectins, soy protein, high protein diets

## Abstract

Both defective and persistent angiogenesis are linked to pathological situations in the adult. Compounds able to modulate angiogenesis have a potential value for the treatment of such pathologies. Several small molecules present in the diet have been shown to have modulatory effects on angiogenesis. This review presents the current state of knowledge on the potential modulatory roles of dietary proteins on angiogenesis. There is currently limited available information on the topic. Milk contains at least three proteins for which modulatory effects on angiogenesis have been previously demonstrated. On the other hand, there is some scarce information on the potential of dietary lectins, edible plant proteins and high protein diets to modulate angiogenesis.

## 1. Introduction

The generation of new blood vessels from pre-existing ones is very important biological process called angiogenesis. During embryonic development, angiogenesis is essential and required for a proper development of a functional circulatory system providing nutrients and oxygen to every cell of the body and warranting waste product removing from all of them. In contrast, angiogenesis is restricted to few processes in the adult, namely, those related to reproductive cycle, bone repair and wound healing. In all these cases, angiogenesis occurs transiently and is highly regulated. On the contrary, there are many pathological situations characterized by (and dependent on) a persistent and deregulated angiogenesis [1,2]. The long list of angiogenesis-dependent diseases includes a number of seemingly unrelated diseases, including psoriasis, endometriosis, proliferative retinopathies, rheumatoid arthritis and many types of cancer. In theses angiogenesis-dependent diseases the so-called angiogenic switch is “on” due to an imbalance of pro-angiogenic regulatory factors over anti-angiogenic ones [1]. On the other hand, there are pathologies such as ischemic diseases where there is an insufficient blood supply. In principle, compounds able to control the “on”/“off” status of the angiogenic switch could be candidates for the pharmacological treatment of angiogenesis-dependent diseases (anti-angiogenic compounds) or for those diseases lacking sufficient blood supply (pro-angiogenic compounds). This explains the great interest maintained for the last 20 years by pharmaceutical industry and research groups looking for modulatory compounds of angiogenesis [3,4,5].

In the past years, food and dietary components have become an important source of natural bioactive compounds with newly identified modulatory effects on angiogenesis. This is the case of genistein, contained in soy [6]; epigallocatechin 3-gallate, abundant in green tea [7]; resveratrol, contained in wine [8]; kahweol, present in unfiltered coffee [9]; oleocanthal and hydroxytyrosol, two constituents of virgin extra olive oil [10,11]; and carnosol and carnosic acid, two major components of rosemary extracts [12], among others. In contrast, little is known regarding diet proteins and their potential involvement in angiogenesis regulation. The aim of this review is to focus on available information in scientific literature regarding this topic. A literature search strategy based on the retrieval of articles containing “dietary protein” as MeSH Major Topic AND the term “angiogenesis” yielded 46 reports, the oldest one dated 1995. A manual curation based on the abstracts contents led to the selection of 21 articles containing valuable information on the topic. Interestingly, three of the currently best characterized dietary proteins as potential modulators of angiogenesis are contained in milk. There is also some limited information regarding dietary lectins, soy proteins and high protein diets.

## 2. Milk Angiogenin-2

**Figure 1:**
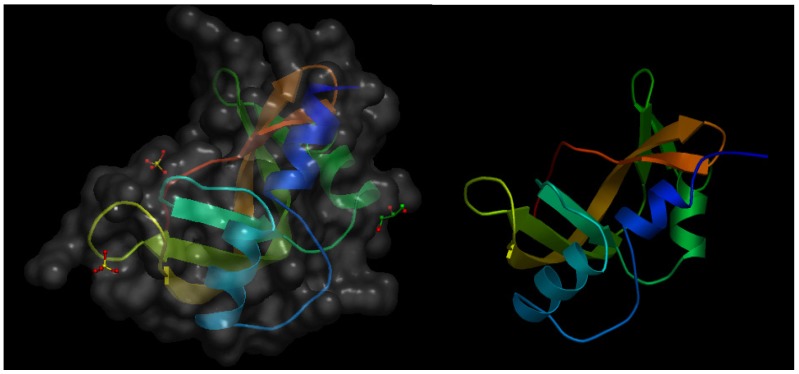
(**Left**) Schematic representation of the crystal structure of murine angiogenin-2 at a resolution of 1.64 Å. The file with pdb ID 3zbv was retrieved from protein data bank [16] and visualized with Open Astex Viewer maintaining the protein on a black background and representing the protein surface in grey with a 30% of opacity. (**Right**) Schematic representation of the available model of bovine angiogenin-2 obtained by homology modeling, based in the crystal structure of bovine angiogenin-1 as a template sharing 58% of sequence identity. The file with psi ID P80929 was retrieved from protein model portal [17] and visualized with Open Astex Viewer maintaining the protein on a black background.

Angiogenin-1 (or p15 protein) is a small protein that behaves as a strong angiogenesis inducer [13] and exhibits RNase activity, which is a prerequisite for its pro-angiogenic effect [14]. Twelve years after the initial characterization of angiogenin-1, the same research group published an article describing the isolation and characterization of angiogenin-2, a protein present in bovine serum and milk and exhibiting an extremely low RNase activity as compared with that of angiogenin-1 [15]. In this article, the authors report the results obtained in the *in vivo* chick embryo chorioallantoic membrane (CAM) assay, clearly showing that bovine angiogenin-2 is a pro-angiogenic protein with an angiogenesis inducing effect as potent as that exhibited by human angiogenin-1. Figure 1 shows the crystal structure of murine angiogenin-2 and a model of bovine angiogenin-2.

## 3. Milk Lactoferrin

**Figure 2:**
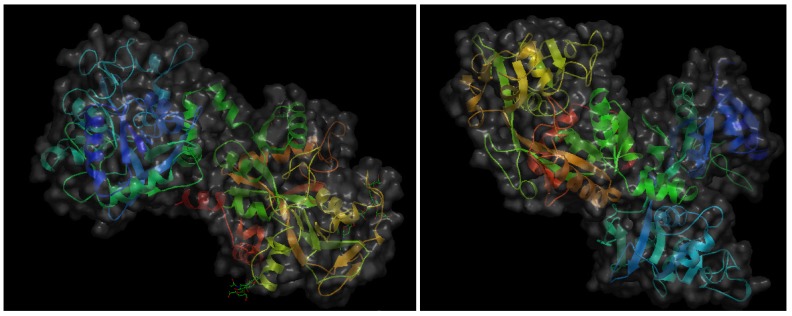
Schematic representation of the 3-D structures of bovine hololactoferrin at a resolution of 2.8 Å (**left**) and human apolactoferrin at a resolution of 2.0 Å (**right**). The files with pdb ID 1blf (bovine hololactoferrin) and 1cb6 (human apolactoferrin) were retrieved from protein data bank [16] and visualized with Open Astex Viewer maintaining proteins on a black background and representing protein surfaces in grey with a 30% of opacity.

The same year 1997 in which milk angiogenin-2 was isolated and characterized as a pro-angiogenic milk protein, a Japanese research group reported that another bovine milk protein, lactoferrin (bLF), and a pepsin-generated peptide of bLF, lactoferricin (bLfcin) were able to inhibit tumor angiogenesis and metastasis in syngenic mice experimental models [18]. LF is a 78–80 kDa mammalian heparin- and iron-binding single-chain glycoprotein that appears in both iron-saturated (holo-LF) and iron-depleted (apo-LF) forms. LF is a major component of the secondary granules in neutrophilic leukocytes, from which it is released during inflammatory responses [19]. Apo-LF is secreted in most exocrine fluids, including tears, milk, colostrum, uterine secretions and semen [20]. Initially described as an iron-binding protein with bactericidal effects [21], currently LF is considered to be a multifunctional protein playing important roles in the control of cell growth and differentiation and exhibiting anti-tumor and anti-inflammatory activities [22,23,24,25,26]. It should be underscored that the anti-angiogenic and anti-metastatic effects reported by Yoo *et al*. [18] was only observed when apo-bLF was used, contrasting with the fact that both holo-bLF and human apo-LF failed to induce this inhibitory effect. Figure 2 shows the crystal structures of both holo-bLF and human apo-LF. Several years later, another Japanese group assessed the *in vivo* anti-angiogenic effects of apo-bLF both in the CAM angiogenesis model and in tumor-induced angiogenesis using the dorsal air sac assay [27]. In this study, *in vitro* anti-angiogenic effects of apo-bLF were also observed in endothelial cell cultures. Namely, apo-bLF inhibited endothelial cell tube formation on Matrigel and proliferation in a dose-response manner. Based on these results, the authors suggest the potential of apo-bLF as an oral therapeutic agent for the treatment of angiogenesis-dependent diseases.

Klas Norrby has made key contributions elucidating the opposed effects of apo-bLF and apo-hLF on angiogenesis [28,29]. On the one hand, in a collaborative work in which he is the first signing co-author the VEGF-A165-mediated angiogenesis in the rat is shown to be effectively inhibited by orally administered apo-bLF [28]. When microvessels were observed and compared in rats treated with VEGF-A165 and receiving either apo-bLF or the vehicle orally, the quantification of the angiogenic response *in vivo* showed that apo-bLF significantly reduced the vascularized area, the total microvascular lenth and the index of microvessel intersection. In contrast, Norrby has shown that apo-hLF orally administered increased VEGF-A165-mediated angiogenesis in rat [29]. Based on these effects, Norrby hypothesized that systemic administration of apo-hLF could be useful for the pharmacological promotion of collateral blood vessel formation to counteract ischemia and infarction [29].

**Figure 3:**
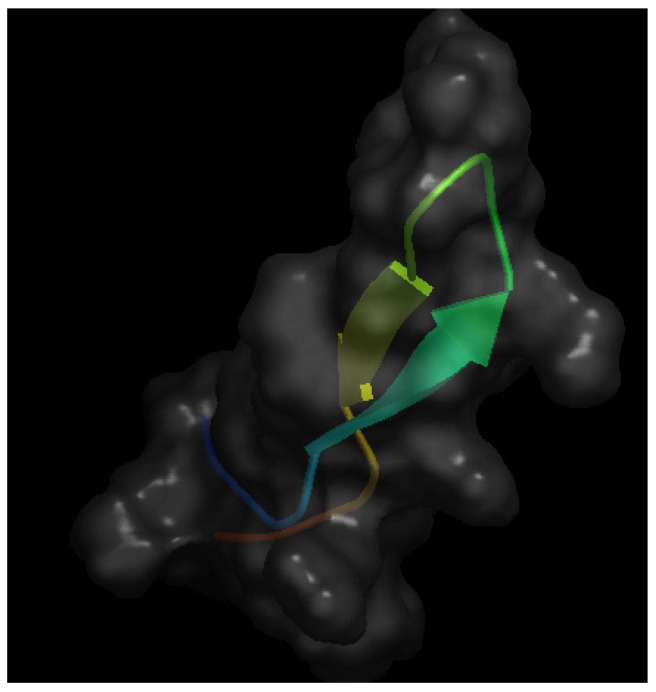
Schematic representation of the 3-D structure of bovine lactoferricin in solution solved by NMR. The file with pdb ID 1lfc was retrieved from protein data bank [16] and visualized with Open Astex Viewer maintaining the protein on a black background and representing the protein surface in grey with a 30% of opacity.

As mentioned above, bLfcin is a pepsin-generated peptide derived from bLF that seems to maintain the anti-angiogenic effect of apo-bLF [18]. Figure 3 shows the crystal structure of bLfcin. Under acidic conditions, pepsin cleaves the *N*-terminal region of bLF, yielding the 25-amino acid cationic peptide bLfcin, which has an amphipathic, anti-parallel β-sheet structure [30]. A Canadian team carried out a deeper study of the anti-angiogenic activity of bLfcin [31]. Using *in vitro* approaches, this study shows that bLfcin binds human umbilical endothelial cells (HUVEC) in a heparin-dependent manner and that this binding can be inhibited by both bFGF and VEGF-A165. Furthermore, it is shown that bLfcin inhibits bFGF- and VEGF-A165-induced HUVEC proliferation and migration without affecting HUVEC viability. Using the *in vivo* Matrigel plug assay, the authors show that bLfcin is able to reduce the pro-angiogenic effects elicited by bFGF and VEGF-A165. Taken together, these results suggest a potential application of bLfcin for the pharmacological treatment of angiogenesis-dependent cancer progression.

Confirming previous results and adding new molecular targets for the anti-inflammatory and anti-angiogenic effects of apo-bLF, a recent study with bovine aortic endothelial cells (BAEC) has shown a number of interesting results: (i) Apo-bLF decreases the adhesion of leukocytes to LPS-activated BAEC; (ii) Apo-bLF inhibits in a dose-dependent manner the LPS-induced ICAM-1, IL-1beta and IL-6 mRNA expression levels in BAEC; (iii) Apo-bLF inhibits in a dose-dependent manner BAEC proliferation, migration and tube formation on Matrigel [32].

Very recently, a study using an *in vivo* lung cancer therapy model has revealed that oral administration of apo-bLF was able to inhibit angiogenesis and to block lung cancer cell inflammation [33].

## 4. Milk Lactadherin

**Figure 4:**
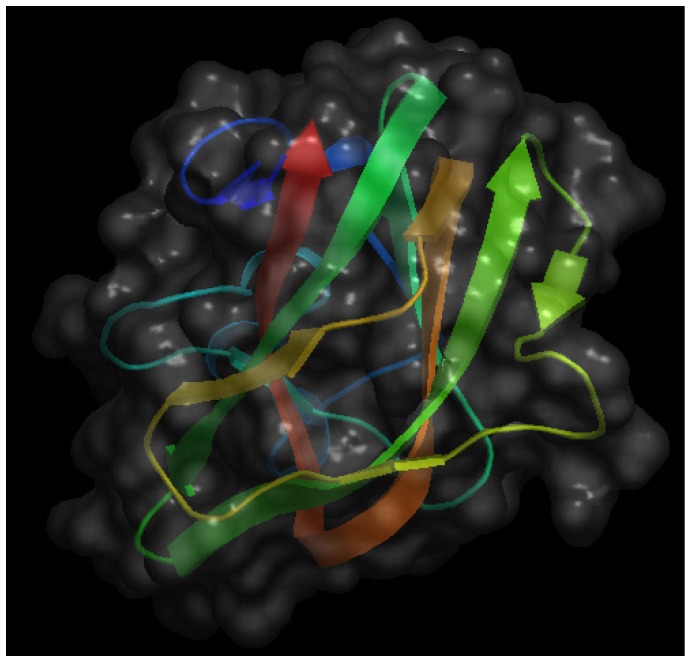
Schematic representation of the crystal structure of the C2 domain of bovine lactadherin at a resolution of 1.67 Å. The file with pdb ID 3bng was retrieved from protein data bank [16] and visualized with Open Astex Viewer maintaining the protein on a black background and representing the protein surface in grey with a 30% of opacity.

A third interesting bio-active protein present in milk is lactadherin. Figure 4 shows the crystal structure of the C2 domain of bovine lactadherin. Also known as MFG-E8 (from Milk Fat Globule Epidermal growth factor-8), it was initially identified as one of the main components of milk fat globules, membrane-surrounded protein- and triacylglycerol-enriched lipid droplets that bud from the apical surface of mammary epithelia during lactation [34,35]. More recently, the acronym SED1 has been proposed as an alternative name for lactadherin, making reference to its nature of being a secreted protein with a multidomain structure, comprising an *N*-terminal domain with one (in human) or two (in mouse and rat) EGF-like domains (the second of which with an integrin-binding RGD motif) and a *C*-terminal domain with two discoidin/F5/8C domains that bind to anionic phospholipids and extracellular matrices. Behaving as an opsonin, lactadherin contributes to phagocytic removal of apoptotic cells in different tissue and facilitates a number of intercellular interactions, recently reviewed in [36].

Interestingly, lactadherin shares structural domain homology with the potent pro-angiogenic protein Del-1 [37], suggesting that lactadherin might be a good candidate for modulation of neovascularization. This suggestion is exhaustively studied in a remarkable article showing that, in fact, lactadherin promotes VEGF-dependent angiogenesis [38]. This study provided a rationale for the evaluation of lactadherin as a potential new target for neoangiogenesis therapeutic modulation.

Some years later, the second coauthor of the previously commented article signed as senior investigator of another study ruling out any major role of the pro-angiogenic effects of lactadherin for its pro-tumor role in the case of bladder carcinoma [39]. On the other hand, other three relevant articles evaluate the potential of lactadherin as therapeutic target using different model systems and experimental approaches [40,41,42]. Neutzer *et al*. [40] made use of Rip1-Tag2 transgenic mice, an experimental model of critically angiogenesis-dependent pancreas cancer, to determine whether lactadherin affects tumorigenesis. By generating lactadherin KO animals in this model, the study reveals that, in fact, lactadherin is critically involved in turning on the angiogenic switch in pancreatic tumorigenesis but that is not requested as essential for angiogenic islets progression to solid tumors. Based on the multidomain structure of lactadherin, the authors of this study suggest producing truncated and stable forms of this protein that could be used as therapeutic agents. The approach used by Fens *et al*. [41] is completely different: based on the fact that lactadherin behaves as an opsonin for the rapid clearance of apoptotic cells and taking into account that tumor angiogenic endothelium expresses integrins to which lactadherin attaches, this study demonstrates that tumor angiogenic endothelial cells have indeed the ability to phagocytose lactadherin-opsonized large particles, aged erythrocytes and apoptotic cells. In this study, the authors also prepare RGD-peptide engineered erythrocytes and discuss their diagnostic and/or therapeutic utility showing their antitumor effects in a murine model of melanoma. Finally, the authors suggest the use of these modified erythrocytes to encapsulate antitumor agents to strengthen their therapeutic impact. Very recently, Jinushi *et al*. [42] evaluate lactadherin as a general target for anti-tumor therapy showing that antibodies directed to lactadherin in combination with conventional anti-tumor treatments enhances drug-induced apoptosis, stimulates T cell immunity, increases dendritic cell cross-presentation of tumor antigens and modulates dendritic cell cytokine production.

Finally, a very recent article shows that secretory phospholipase A_2_ activity is increased on pre-apoptotic leukemia cells and that lactadherin inhibits this activity, thus opening another way for potential uses of lactadherin, or lactadherin-like molecules [43].

## 5. Lectins

Lectins are proteins or glycoproteins with potent biological activity characterized by their ability to agglutinate erythrocytes of some or all blood groups *in vitro* [44]. Due to their potent bioactivity, some lectins are among the most powerfully cytotoxic compounds known, as is the case of ricin and mistletoe lectins. However, many other lectins are non-toxic, since they are ubiquitous in our food supply. Lectins are especially abundant in legumes, but they are also present in cereals, tomato, potato, mushroom and banana, among others [45]. Lectins have been shown to resist gastrointestinal digestion [46] and to maintain full biological activity after entering the circulatory system. A review has summarized the published scientific evidence in favor of anti-tumor mechanisms of action of lectins [45]. One of the studies mentioned in this review is that of Park *et al*. [47], showing that the Korean mistletoe agglutinin VCA (*Viscum album* L. var. *coloratum*) increases tumor cell apoptosis and inhibits tumor angiogenesis, thus leading to a decreased rate of metastasis. Currently, there is no available scientific information on the potential modulatory role of food lectins on angiogenesis but the findings related VCA should be a stimulus for future investigations on the topic.

## 6. Soy Proteins

It is known that the maintenance of adipocyte functionally in obesity is related to induced angiogenesis and extracellular matrix remodeling activities to warrant blood supply. In a recent study, it has been shown that soy proteins attenuate abnormalities of the renin-angiotensin system in adipose tissue from obese rats maintaining adipocyte functionality through a stimulation of angiogenesis [48]. This initial observation requires to be further investigated.

## 7. High Protein Diet

Since obesity and overweight are related to increased incidence of many pathologies and these conditions have been pandemic in Western countries, currently there is a high demand of more effective and safe dietary approaches for weight loss. The DIOGENES (Diet, Obesity, and GENES) trial has shown that better weight maintenance is achieved with the combination of a modest increase in protein content and a reduction in the glycemic index of the diet [49]. Based on this information, a recent nutritional study evaluated whether an energy-restricted high-protein diet with a low glycemic index and soluble fiber would be more effective than a conventional low-calorie diet for weight loss and related metabolic risk factors [50]. This is a randomized controlled trial that enrolled only 8 men and 5 postmenopausal women to make affordable a systemic approach to evaluation by using not only a follow up of conventional variables but also post genomic and systems biology procedures, including microarray gene expression analysis and the use of potent statistical and network analysis. The study carries out a comprehensive analysis of the gene expression patterns associated with adipocyte size during the entire dietary weight-loss intervention. The systemic analysis of the results shows that the high protein diet with a low glycemic index and soluble fiber orchestrates a coordinated program of gene expression involving increases in molecules that enhance apoptosis and inhibit adipogenesis, cell migration, adhesion and angiogenesis.

## 8. Conclusions

In this review, we have shown that several dietary proteins are able to modulate angiogenesis and that this fact opens new avenues for therapeutic interventions. It is remarkable that three proteins already characterized as modulators of angiogenesis are present in milk. Nonetheless, the overall picture depicted by this review is that systemic searches for dietary proteins able to modulate angiogenesis remain to be carried out. In this research area, new discoveries and advancements towards their application in therapeutic settings are expected for the near future.
